# Dexamethasone-Mediated Upregulation of Calreticulin Inhibits Primary Human Glioblastoma Dispersal Ex Vivo

**DOI:** 10.3390/ijms19020572

**Published:** 2018-02-14

**Authors:** Mohan Nair, Juan Romero, Aria Mahtabfar, Ahmed M. Meleis, Ramsey A. Foty, Siobhan A. Corbett

**Affiliations:** 1 Department of Surgery, Rutgers Robert Wood Johnson Medical School, One Robert Wood Johnson Place, New Brunswick, NJ 08901, USA; mohanrbk@rwjms.rutgers.edu (M.N.); romeroju@scarletmail.rutgers.edu (J.R.); am1823@rwjms.rutgers.edu (A.M.); corbetsi@rwjms.rutgers.edu (S.A.C.); 2 Department of Neurological Surgery, Rutgers New Jersey Medical School, 90 Bergen Street, Newark, NJ 07101, USA; meleisah@njms.rutgers.edu

**Keywords:** dexamethasone, calreticulin, glioblastoma, dispersal, inhibition, retina model, brain slices

## Abstract

Dispersal of Glioblastoma (GBM) renders localized therapy ineffective and is a major cause of recurrence. Previous studies have demonstrated that Dexamethasone (Dex), a drug currently used to treat brain tumor–related edema, can also significantly reduce dispersal of human primary GBM cells from neurospheres. It does so by triggering α5 integrin activity, leading to restoration of fibronectin matrix assembly (FNMA), increased neurosphere cohesion, and reduction of neurosphere dispersal velocity (DV). How Dex specifically activates α5 integrin in these GBM lines is unknown. Several chaperone proteins are known to activate integrins, including calreticulin (CALR). We explore the role of CALR as a potential mediator of Dex-dependent induction of α5 integrin activity in primary human GBM cells. We use CALR knock-down and knock-in strategies to explore the effects on FNMA, aggregate compaction, and dispersal velocity in vitro, as well as dispersal ex vivo on extirpated mouse retina and brain slices. We show that Dex increases CALR expression and that siRNA knockdown suppresses Dex-mediated FNMA. Overexpression of CALR in GBM cells activates FNMA, increases compaction, and decreases DV in vitro and on explants of mouse retina and brain slices. Our results define a novel interaction between Dex, CALR, and FNMA as inhibitors of GBM dispersal.

## 1. Introduction

Glioblastoma remains an intractable disease due to its propensity for early dispersal. Despite surgical resection of the tumor mass followed by radiotherapy and chemotherapy, glioblastoma always recurs and ultimately leads to patient death. Few strategies have been devised to control this aggressive behavior. Altering tumor behavior to keep cells “in place” may make targeted therapy for recurrence more effective.

Previous studies have shown that Dexamethasone, a drug currently used to treat brain tumor related edema, is very effective in not only inhibiting dispersal of primary human GBM neurospheres but can also reduce their growth rate. Inhibition of dispersal was demonstrated to be largely due to a Dex-mediated activation of α5 integrin function and subsequent reactivation of fibronectin matrix assembly (FNMA), a process that in a 3D neurosphere “glues” cells together, effectively inhibiting cell detachment from the primary mass. Integrin activation also results in a significant re-localization of cortical actin into stress fibers, changes in cell morphology, stronger attachment to substrate, and decreased motility. The combination of increased intercellular cohesion and decreased motility significantly reduces the dispersal velocity of GBM neurospheres [1]. How Dex specifically activates α5 integrin in these GBM lines is unknown.

Several chaperone proteins are known to activate integrins, including talin [2], kindlin-1 and kindlin-2 [3], and calreticulin (CALR) [4,5]. In this study, we focus on CALR, since several studies have revealed a connection between disruption of CALR function and various malignancies including myeloproliferative disorders [6], osteocarcoma [7], ovarian and non-small cell lung cancer [8], breast cancer [9], and Glioblastoma (GBM) [10]. CALR was first identified in 1974 by Ostwald and MacLennan [11] and has been extensively studied for over 40 years. CALR is an endoplasmic reticulum (ER) chaperone protein, and when localized to the ER, its role is to direct proper folding of proteins and glycoproteins and to maintain homeostatic control of cytosolic and ER calcium levels [12]. Mounting evidence now suggests that CALR can localize to the outer cell surface [13], to the cytosol [14], and even to the extracellular matrix (ECM) [15]. Accordingly, its role has now expanded to control key biological processes associated with wound healing [16], the immune response [17], fibrosis [18], and cancer [19]. Moreover, in cell lines that are CALR deficient, addition of exogenous CALR rescues various CALR-driven processes such as cell adhesion and migration [20]. This suggests that CALR may either be directly or indirectly involved in modulating molecular mechanisms associated with such processes.

Moreover, TCGA [21] analysis for CALR expression in both GBM and low-grade gliomas (LGG) showed that of the 591 sequenced GBM samples, only three (0.5%) patients demonstrated genetic alteration of CALR [22,23]. All three patients exhibited amplification of the gene. These findings suggest CALR does not play a prominent role in tumorigenesis. However, those patients with over-expression of CALR demonstrated an increase in median survival time of 28.91 months compared to 14.13 months in patients with non-upregulated CALR. These findings suggest a correlation between higher CALR expression and overall survival; however due to the relatively small sample size (three patients) it is difficult to determine rigorous statistical correlation. Similarly, low-grade gliomas (LGG) demonstrated only 11 mutations (1.9%) in CALR in the 591 patient samples tested. In LGG, survival between mutated CALR and non-mutated CALR were relatively similar, 12.61 months vs 14.19 months, respectively. Based on a review of the TCGA data, it appears that while not prognostic, amplification of CALR appears to correlate with longer median survival.

**Figure 1:**
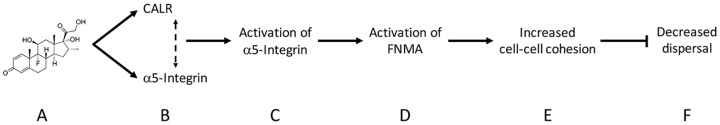
Scheme of working hypothesis. Dexamethasone (**A**) treatment leads to the upregulation of α5 integrin and calreticulin (CALR), a chaperone protein for α5-integrin that upregulates its activity either by direct interaction [4], or indirectly by phosphorylation/dephosphorylation of regulatory proteins [5] (**B**). Activation of α5-integrin (**C**) leads to a significant increase in capacity for fibronectin matrix assembly (FNMA) (**D**), effectively “gluing” cells together. We hypothesize that restoration of FNMA by CALR will result in an increase in the strength of cell-cell cohesion (**E**), decreased cell detachment and reduced dispersal of Glioblastoma (GBM) cells (**F**). A decrease in dispersal could, in principle, keep the recurrent tumor more contained, thus rendering it amenable to localized therapy.

Given CALR’s demonstrated role in integrin activation, in influencing adhesion and migration, and its potential role in improving survival, we asked whether CALR may be involved in Dex-mediated activation of integrin function in these GBM lines. We first determined whether simply overexpressing α5 integrin in GBM cells is sufficient to activate FNMA. We then assessed CALR levels in these lines and whether Dex-treatment increased CALR expression. We explored the effects on FNMA of siRNA knockdown of CALR in Dex-treated GBM cells. We then determined whether overexpression of CALR was sufficient to activate FNMA in GBM. Finally, we assessed the effects of CALR overexpression on neurosphere cohesion, and on dispersal in vitro and in an ex vivo mouse retina and brain slice assay. The overarching hypothesis tested here is that expression of CALR in deficient GBM-cells is sufficient to restore FNMA and that this will result in an increase in the strength of cell-cell cohesion, decreased cell detachment and reduced dispersal (Figure 1).

## 2. Results

### 2.1. Overexpression of α5 Integrin Fails to Induce FNMA in GBM Cells

**Figure 2:**
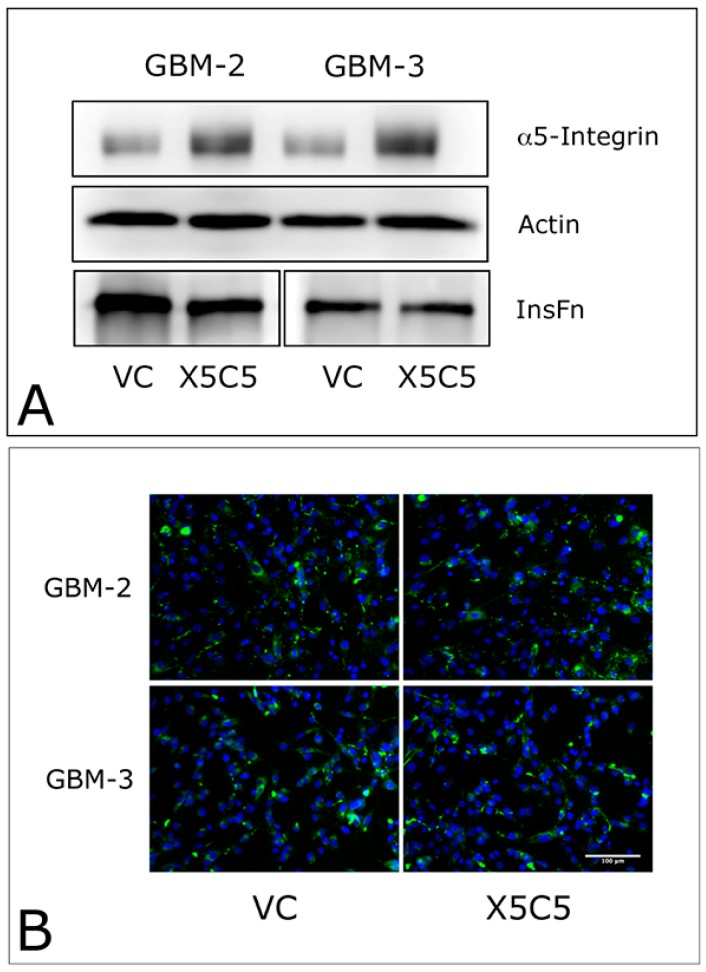
Overexpression of α5 integrin fails to promote FNMA in GBM cells. Immunoblot (**A**) and immunofluorescence (**B**) analysis of FNMA by integrin (X5C5) transfected GBM-2 and GBM-3 cells shows no appreciable increase in the production of an insoluble fibronectin matrix (InsFn) relative to empty vector controls (VC). Green signal (assembled fibronectin matrix), blue signal (DAPI counterstain).

Previous studies from our lab demonstrated that Dex treatment upregulates α5 integrin expression and fibronectin matrix assembly, leading to increased tumor cohesion and decreased dispersal [1]. Therefore, it is logical to assume that overexpression of α5 integrin may be sufficient to upregulate FNMA. To establish causality, we transfected GBM-2 and GBM-3 cells with an expression vector for α5 integrin (X5C5) or an empty vector control (VC). Figure 2 shows that overexpressing α5 integrin did not result in increased FNMA, as demonstrated by no change in the abundance of insoluble fibronectin (InsFn), as demonstrated by immunoblot (Figure 2A) or immunofluorescence (Figure 2B) assays.

### 2.2. Dexamethasone Up-Regulates CALR Expression by GBM Cells

**Figure 3:**
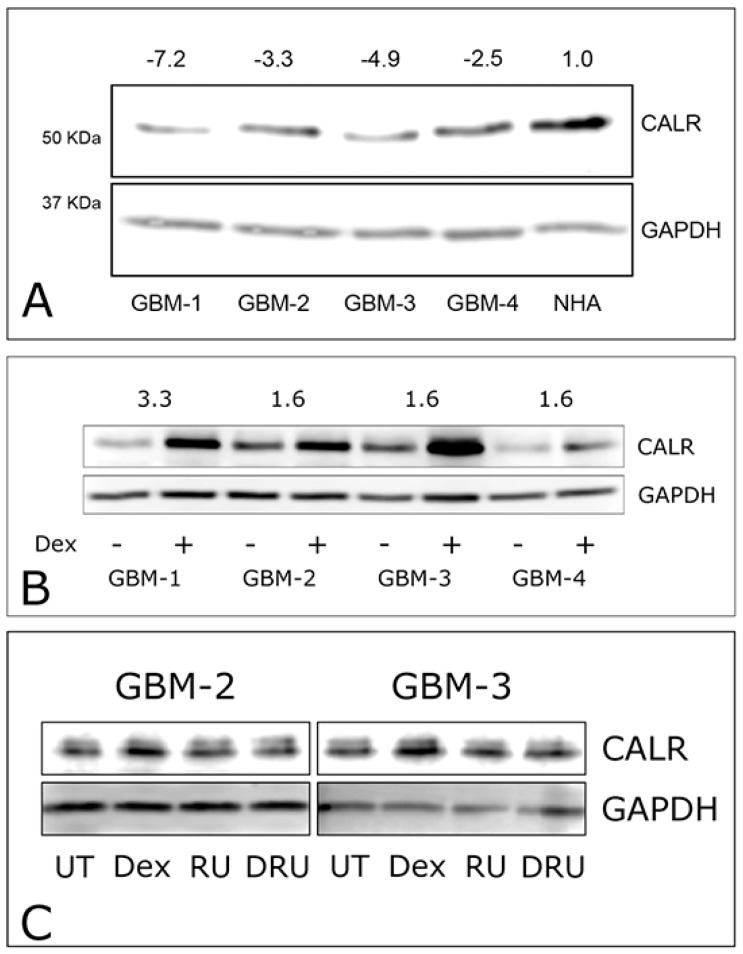
CALR expression and modulation by Dexamethasone. Immunoblot analysis reveals that CALR expression is 2- to 7-fold lower in GBM cells than in normal human astrocytes (**A**). CALR expression is significantly up-regulated when cells are incubated for 24 h in 1 × 10^−7^ M dexamethasone relative to vehicle only controls (**B**). GBM cells treated with Dex show higher levels of CALR expression. Cells incubated in the corticosteroid receptor antagonist RU-486, (RU) or in a combination of Dex and RU-486 (DRU) show similar levels of CALR expression as untreated (UT) cells (**C**).

CALR is a key mediator of α5 integrin activity and could, in principle, be involved in regulating FNMA. First, we compared the levels of CALR expression in GBM cells relative to those of normal human astrocytes (NHA). Immunoblot analysis reveals that CALR expression by GBM cells is 2 to 7-fold lower than those expressed by NHA cells (Figure 3A). We then asked whether CALR levels are affected by Dexamethasone treatment. Interestingly, Dex treatment resulted in a 1.6 to 3-fold increase in CALR relative to untreated controls (Figure 3B). We compared densitometry data of CALR expression for untreated (*n* = 4) and Dex treated (*n* = 4) samples of GBM-1–4. Analysis by Wilcoxon Rank-Sum test generated a *p*-value of 0.0416, confirming that Dex treatment significantly increases CALR expression in GBM cells. Previous studies showed that the corticosteroid receptor inhibitor, RU-486, abolishes Dex-mediated induction of FNMA in GBM cells [1]. Here, we show that RU-486 also blocks Dex-mediated upregulation of CALR (Figure 3C). Collectively, these results suggest that Dex not only regulates the expression of α5 integrin, but also of CALR, a key regulator of its activity.

### 2.3. CALR siRNA Blocks Dex-Mediated Up-Regulation of FNMA

**Figure 4:**
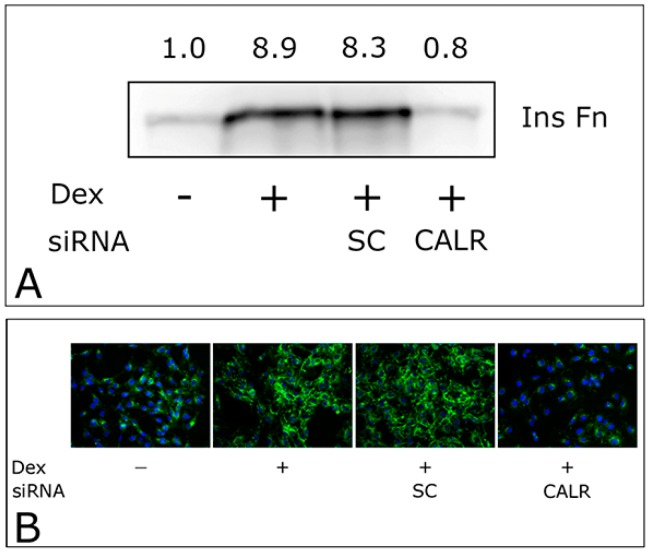
CALR siRNA blocks FNMA in dexamethasone treated GBM cells. Immunoblot (**A**) and immunofluorescence (**B**) assays demonstrating the effects of CALR siRNA on FNMA in GBM cells. Note the significant reduction in the production of an insoluble fibronectin matrix (InsFn) in the presence of the CALR siRNA relative to a scrambled control (SC). Green signal (assembled fibronectin matrix), blue signal (DAPI counterstain).

To further explore the connection between Dex, CALR and FNMA, we performed experiments in which FNMA was assessed in GBM cells that were transfected with either a CALR siRNA or scrambled control (SC) prior to Dex treatment. Figure 3 shows that CALR siRNA almost completely abolishes FNMA in Dex-induced GBM cells when assessed by both immunoblot (Figure 4A) and immunofluorescence (Figure 4B) analysis.

### 2.4. CALR Transfection Promotes FNMA by GBM Cells

**Figure 5:**
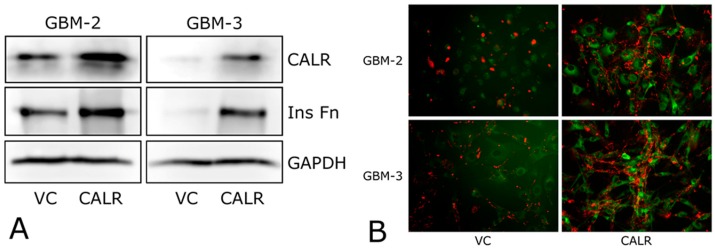
Transfection of GBM cells with a CALR expression vector induces FNMA. Immunoblot (**A**) and immunofluorescence (**B**) analysis of GBM-2 and GBM-3 cells transfected with a CALR expression plasmid or an empty vector control (VC). Increased levels of CALR correspond to an increase in the production of an insoluble fibronectin matrix (InsFn, A) and in long fibronectin fibrils (**B**). Red signal (assembled fibronectin matrix), green signal (CALR-GFP fusion protein).

We next assessed whether increased CALR expression is sufficient to induce FNMA in GBM cells. GBM-2 and GBM-3 cells were transfected with either a vector control (VC) or a CALR expression plasmid. Transfection with the CALR plasmid resulted in a marked increase in CALR expression and in the assembly of insoluble fibronectin relative to VC controls (Figure 5A). Assessment of FNMA by immunofluorescence also showed a significant assembly of fibronectin into long fibers extending between cells (Figure 5B).

### 2.5. Overexpression of CALR Significantly Reduces Dispersal of GBM Cells

**Figure 6:**
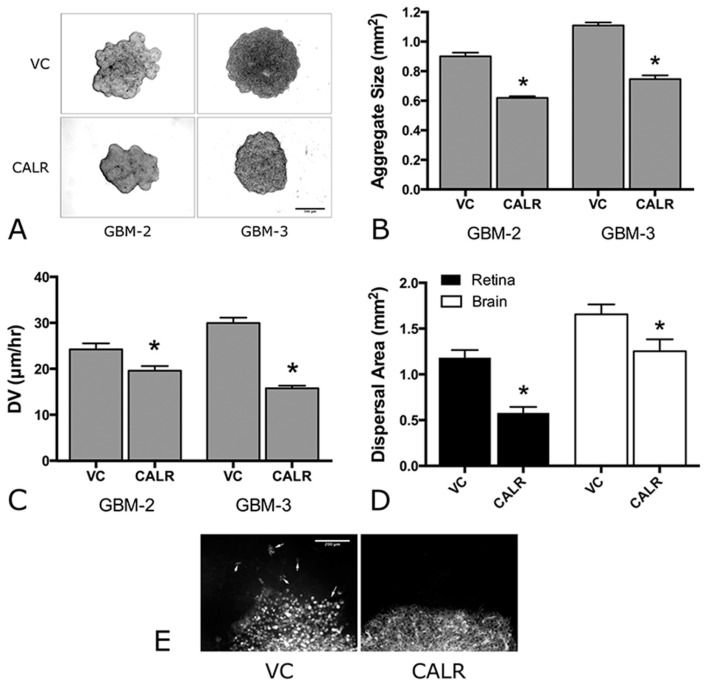
Effects of CALR expression on GBM dispersal. Compaction assay of GBM-2 and GBM-3 cells transfected with CALR plasmid (CALR) or empty vector control (VC). CALR appears to result in smaller aggregates (**A**). Quantification of CALR mediated compaction (**B**). Mean size and standard error for *n* = 10 aggregates of GBM-2 and GBM-3 after 48 h in culture. Asterisks represent statistical difference by pair-wise comparison and Student’s *t*-test, *p* < 0.0001). Measurement of dispersal velocity (**C**). The dispersal velocities of CALR transfected aggregates of GBM-2 and GBM-3 (*n* = 31 and *n* = 28, respectively) was significantly lower than that measured for control aggregates (*n* = 28 and *n* = 31). Asterisks represent significant difference at *p* < 0.05 by pair-wise comparison using Student’s *t*-test. Ex vivo retina dispersal assay (**D**). Dispersal of GBM aggregates on extirpated mouse retinas and on mouse brain slices was quantified by placing aggregates of fluorescently labeled control (*n* = 12) or CALR-transfected (*n* = 12) GBM-2 cells on mouse retinas and brain slices and incubation under tissue culture conditions for 24 h. Asterisk represents significant difference by Student’s *t*-test, *p* < 0.0001. Representative image of aggregate spreading by control and CALR aggregates of GBM-2 on mouse retina. Note single cell dispersal from control aggregates (white arrows, VC), in contrast to the higher level of cell-cell contact at the advancing cell front observed for CALR aggregates (**E**). Signal in panel E (fluorescent, membrane intercalating dye, PKH67).

To determine whether CALR overexpression and attendant increase in FNMA are able to reduce dispersal, we performed compaction assays to measure changes in aggregate cohesion, and dispersal assays to quantify changes in dispersal velocity. We show in Figure 6 that aggregates of GBM-2 CALR and GBM-3 CALR are more compact than those transfected with the empty vector (VC) control (Figure 6A). This was confirmed by quantitative compaction assays in which aggregate size was compared between vector control and CALR-transfected cells. CALR-transfected aggregates were found to be significantly smaller (0.62 ± 0.01 and 0.75 ± 0.02 mm^2^) than control aggregates (0.90 ± 0.02 and 1.11 ± 0.02 mm^2^) for GBM-2 and GBM-3, respectively (Figure 6B), as determined by pair-wise Student’s *t*-test at *p* < 0.05). To determine whether increased compaction results in a decrease in dispersal, two assays were employed. The first measures dispersal velocity of 3D aggregates on tissue culture plastic. Figure 6C shows that the DV of aggregates composed of CALR-transfected cells is significantly slower (19.61 ± 0.99 and 15.78 ± 0.56 μm/h) than those transfected with vector controls (24.25 ± 1.29 and 29.98 ± 1.16 μm/h), respectively, when compared by pair-wise Student’s *t*-test at *p* < 0.05. We also performed dispersal assays in which aggregates of GBM-2 cells were plated either onto extirpated mouse retinas or on mouse brain slices. In both cases, the extent of dispersal was much lower for CALR aggregates than for control aggregates when compared by Student’s *t*-test (*p* < 0.0001 for retinas and *p* = 0.032 for brain slice, Figure 6D). Moreover, the pattern of dispersal was also markedly different. Whereas, the advancing edge of control aggregates dispersed as single cells (Figure 5E, left panel), the leading edge of GBM-2 aggregates composed of CALR-transfected cells advanced as a cohesive sheet (Figure 6E, right panel), further confirming that CALR, by inducing FNMA, can give rise to decreased dispersal of GBM cells.

## 3. Discussion

Previous studies in which Chinese Hamster Ovary (CHO) cells were transfected to express α5 integrin demonstrated a remarkable restoration of fibronectin matrix assembly [24,25]. In conventional 2D culture, cells utilize fibronectin as a source of cell-ECM adhesion. In 3D culture and in tissues, however, this fibronectin matrix can effectively “glues” cells together by indirectly linking cells through their integrins and the peri-cellular fibronectin matrix. Accordingly, increased FNMA has been demonstrated to significantly increase the cohesion of 3D spheroids [24,25]. Increased aggregate cohesion significantly reduces detachment of cells from a primary mass [26] and effectively inhibits dispersal of single cells. However, these studies employed a genetic approach to increase cohesion. From a practical perspective, a pharmacologic approach to enhance cohesion may be preferable.

Interestingly, restoration of FNMA could also be achieved by treating certain cell lines with Dexamethasone. HT-1080 human fibrosarcoma cells and primary human GBM lines, for example, are deficient in formation of fibronectin matrix fibrils but assembly can be induced by Dex [1,27]. Interestingly, restoration of FNMA in GBM cells is associated with an increase in the expression of α5 integrin [1]. We therefore asked whether GBM cells could be induced to assemble a fibronectin matrix simply by increasing α5 integrin expression, as was observed in CHO cells. This was not the case, suggesting that Dex treatment, irrespective of its regulatory effects on α5 integrin expression, must induce other molecular pathways required for restoration of FNMA. Given that Dex is likely to modulate many pathways, we opted to explore those that have been directly linked to integrin function [28]. We focused on the role of CALR since studies have revealed a connection between altered CALR expression and malignant transformation in a variety of cells, including GBM [10].

To our knowledge, no studies have explored the connection between Dex treatment, CALR expression, and FNMA in GBM cells. Indeed, CALR expression in the 4 primary GBM lines was shown to be, on average, 4-fold lower than that of normal human astrocytes. Moreover, Dex treatment resulted in a significant increase in CALR expression by GBM cells, an effect which could be blocked by the corticosteroid inhibitor, RU-486. A correlation between CALR expression and tumorigenesis has been studied in a variety of cancers. CALR expression has been reported to be both significantly higher in breast [9], pancreatic [29] and lung cancers [8] and significantly lower in prostate tumor tissue [30] when compared to normal prostate. In both instances, altered CALR expression has been associated with correspondingly higher rate of metastases and poor overall survival. Given the expanding role of CALR in a variety of important cell processes, including protein chaperoning, calcium homeostasis and RNA stabilization, it is not surprising that the role of CALR in malignant transformation will vary according to the cell of origin and the molecular subclass of the tumor. In our study, we found diminished CALR in all primary GBM cells that we examined, when compared to normal human astrocytes. That amplification of the CALR gene in a small subset of patients identified in the TCGA database correlated with improved survival, also suggests that Dex-mediated up-regulation of CALR could, in principle, be beneficial.

To further gain insight into the connection between Dex treatment, CALR expression and FNMA in GBM cells, we asked whether increasing CALR expression, in the absence of Dex, is sufficient to restore α5 integrin function and FN matrix assembly. This was indeed the case since transfection of untreated GBM cells with a CALR expression vector resulted in a significant increase in FNMA as detected by both immunofluorescence and immunoblot assays. These data suggest that an increase in CALR expression in these CALR deficient cells is by itself sufficient to promote integrin activation.

In order to determine a functional role for CALR, we transfected GBM cells with either CALR-siRNA or a scrambled control vector and treated them with Dex. We then assessed effects on FNMA. CALR-siRNA completely abolished formation of a fibronectin matrix in Dex-treated cells. These results suggest that Dex-mediated upregulation of CALR may be involved in restoration of FNMA. They do not, however, demonstrate that upregulation of CALR expression is sufficient for restoration of the process.

Several studies have linked calreticulin expression to integrin function. For example, integrin-mediated adhesion is severely impaired in calreticulin-null cells, although cell surface integrin expression is unchanged [31]. As noted above, we observed a similar effect in GBM cells transfected directly with α5 integrin. Despite robust α5 expression, GBM cells still lacked functional α5 as assessed by their ability to assemble FN into insoluble fibers, demonstrating that α5, while necessary for FNMA in GBM cells is not sufficient to establish normal integrin function. Studies have implicated different cellular mechanisms by which CALR may support normal α5 integrin function. One role may involve the process by which CALR regulates calcium homeostasis. In wild-type fibroblasts, engagement of surface integrins induces a transient elevation in cytosolic calcium concentration. When CALR is absent, this calcium influx is lost and seems to correlate with loss of integrin function [31]. However, in this experimental model, there was no alteration in the amount of calcium in endomembrane stores. In addition to its role as a protein chaperone in the ER CALR has been detected in association with cell surface proteins, including integrins [32]. Calreticulin has been detected by immunoprecipitation of surface-biotinylated proteins in platelets, a result confirmed by flow cytometry [33]. Moreover, this investigation demonstrated that antibodies against CALR were shown to inhibit platelet-mediated adhesion. Therefore, it is possible that the effect of CALR may occur via direct binding to the integrin α5, and this is a possibility that we are currently exploring. Alternately, CALR expression may modulate the integrin function via an indirect process. For example, one recent study has linked CALR to the expression of fucosyltransferase, an enzyme which post-translationally modifies the β1 integrin subunit by catalyzing its *N*-glycosylation [34]. This modification is required for normal integrin function. In these experiments, decreased CALR altered integrin activation status, with little effect on integrin expression, suggesting an alternate explanation for this observation in GBM cells.

We then addressed whether CALR-mediated activation of FNMA is sufficient to elicit the dispersal inhibition exhibited by Dex-treated spheroids. We first determined whether CALR expression results in more compact spheroids, a process associated with increased aggregate compaction and cohesion in various cell lines [24,25,35,36]. Interestingly, whereas CALR expressing spheroids of GBM-2 and GBM-3 compacted to approximately 75% of the size of untreated spheroids, the parent GBM-2 and GBM-3 lines when treated with Dex compacted to approximately 38% of untreated spheroids. Dex treatment thus appears to be more effective at promoting compaction than CALR expression alone. This is consistent with the fact that Dex not only upregulates FNMA in GBM cells, but also results in the reorganization of actin into stress fibers. This likely contributes to cell and aggregate compaction due to increased contraction at both the cellular and aggregate levels [1].

We next asked whether CALR expression, significantly decreases dispersal velocity on tissue culture plastic as was observed for aggregates of Dex-treated GBM cells. This also proved to be the case. However, unlike compaction, CALR expression appears to reduce DV equally as effectively as Dex treatment of the parent lines [1]. We also explored whether CALR expression alone could inhibit dispersal of GBM cells on two neural substrates, namely, mouse retina and brain slices. The retina offers several advantages. GBM cells appear to disperse as chords of cells on mouse retina, pattern similar to that observed when GBM cells are injected into mouse brains [37]. Moreover, retina is essentially neural tissue, thus, interactions between retina and GBM will approximate the cell-substratum interactions experienced in vivo. The cellular architecture of the retina, however, is not precisely that of the brain. Accordingly, by comparing dispersal on mouse retina and mouse brain slices, we explored whether the retina is a good substitute for brain tissue. We show that CALR expression can significantly reduce dispersal on both substrates relative to empty vector controls. We also show that the pattern of dispersal is fundamentally different for the CALR expressing cells, the leading edge of CALR-transfected cells advancing as a sheet, rather than as single cells as can be observed in vector-only controls. For a summary of the effects of Dex on the kinetics of CALR, InsFn (FNMA) and Integrin-α5 expression, as well as on cohesion and dispersal, see Table 1.

In order to disperse, GBM cells must first detach from the primary or recurrent mass and establish an optimal adhesion to substrates over which they must locomote. Accordingly, mechanisms involved in cell-cell cohesion, cell-ECM adhesion, and cell motility may be influenced by CALR. That transfection of CALR into GBM cells resulted in restoration of FNMA with an attendant increase in neurosphere compaction and reduced dispersal strongly suggests that an increase in cell-cell cohesion is at least partially responsible for inhibiting cell detachment and dispersal by effectively “gluing” cells more tightly to one another. As such, an increase in FNMA would act as an indirect cell-ECM-cell cohesion mechanism. Activation of α5 integrin can also increase the strength of cell-ECM adhesion. In some cell types, this has been demonstrated to increase migration [38], while in others, the opposite has been shown to be the case [1,39].

It is possible that CALR expression may also influence innate migration of GBM cells. We did not directly test this, although in some cells, glucocorticoids have been shown to increase α5 integrin expression by inhibiting ERK signaling, thus reducing cell migration and invasion [39].

Single cell locomotion is largely dependent on cell shape. If by activating a5 integrin CALR also indirectly changes cell shape to inhibit scattering [40], it is possible that a reduction in capacity for dispersal could further inhibit cell migration through a mechanism of contact inhibition of locomotion (CIL) [41]. In growing colonies, CIL leads to a slowdown of the motility of individual cells when the density of their environment crosses a certain threshold [42]. Different factors can affect collective migration, including crowding (cell density), cohesion (strength of adhesions), and physical constraints [40]. Our results suggest that CALR, by promoting FNMA, may increase the strength of intercellular cohesion, thereby promoting sheet formation, and triggering CIL. Accordingly, it is likely that CALR inhibits GBM dispersal by activating mechanisms that both increase cell-cell cohesion while also decreasing migration. Ours is the first demonstration of an interaction between Dex, CALR, and FNMA as inhibitors of GBM dispersal.

We performed these studies on four low-passage, primary GBM cell lines whose mechanical and molecular properties could be induced by Dex resulting in increased FNMA and reduced dispersal [1]. The gain and loss of function assays were conducted in two of the lines that we previously demonstrated to be more dispersive. Based on the relatively small number of lines tested, we cannot generalize our findings. However, we have previously demonstrated Dex-mediated dispersal inhibition in widely-used GBM lines including, U87MG, LN229, and U118MG [36]. These lines, and others (T98G and U138) were established decades ago and are of incalculable passage number. It is unclear as to whether data generated in such lines are indeed comparable to those generated in low-passage primary cells. We are currently generating more primary cell lines for in vivo testing.

Dex is routinely used as palliative in the therapy of glioblastoma. However, it is widely held that corticosteroid use, particularly concomitant with radiation and chemotherapy, compromises survival of glioblastoma patients [43,44]. This is why, following surgery for resection of GBM, patients are first started on high-dose Dex but are rapidly tapered off the drug over a few weeks [45]. Steroids are not typically prescribed again until after a patient has received chemotherapy and radiation and demonstrate radiographic recurrence on MRI or new neurological deficits. After completing standard chemotherapy and radiotherapy, patients are left with limited therapeutic options to delay time to tumor recurrence and increase progression-free survival. One possible therapy is the use of low-dose Dex [46] to increase CALR and FMNA, resulting in decreased GBM cell dispersal. Keeping the recurrence contained may make it more amenable to localized therapy.

## 4. Materials and Methods

### 4.1. Cell Lines

The GBM-2 and GBM-3 cell lines used in this study were originally generated with approval of the Rutgers-Robert Wood Johnson Medical School Institutional Review Board under protocol #CINJ 001208, approved on 17 March 2017. Samples were anonymized, and the IRB waived the need for written informed consent. The lines used in this study have previously been published and characterized in [1,47]. These lines were previously demonstrated to be highly dispersive. Cells were propagated in Earle’s minimal essential medium containing l-glutamine (ThermoFisher Scientific, Grand Island, NY, USA) and 10% fetal bovine serum (Atlanta Biologicals, Flowery Branch, GA, USA). Cells were cultured under standard tissue culture conditions of 37 °C, 5% CO_2_ and 95% humidity. Final concentration of 0.1 µM dexamethasone (Cat#D2915, Sigma-Aldrich, St. Louis, MO, USA) and or 1 µM RU-486 (cat#ab120356, Abcam, Cambridge, MA, USA) were used for treatment.

### 4.2. Assessment of Fibronectin Matrix Assembly

FN matrix assembly using deoxycholate (DOC) differential solubilization protocol and Western blot analysis was performed as described previously [48]. DOC insoluble lysates probed with a polyclonal anti-FN antibody (ab6584, Abcam, Cambridge, MA, USA) under reducing conditions resolved a 220-kDa band of FN. Soluble lysates from the same samples were probed for either CALR (anti-calreticulin polyclonal antibody cat#06-661, EMD Millipore Corporation, Temecula, CA, USA) and or α5 integrin (Ab1928, Abcam, Cambridge, MA, USA). GAPDH (anti-GAPDH mouse monoclonal antibody, Cat#AM4300, Life Technologies, Waltham, MA, USA) or Actin (anti-Actin, Sigma-Aldrich, St. Louis, MO, USA) were used as loading controls. Western Blot detection was done using Western Bright ECL reagent (BioExpress, Kaysville, UT, USA). Images were captured using a C-Digit blot scanner, and imaging data was analyzed by Image studio software (LI-COR Biosciences, Lincoln, NE, USA).

### 4.3. Immunofluorescence Microscopy

Here, 5 × 10^6^ cells were plated onto 24 well culture dish in complete MEM medium to obtain 80–90% confluence at 24 h. Cells were fixed with 4% paraformaldehyde for 15 min. at room temperature, blocked using CAS-Block (Thermo Fisher Scientific, Grand Island, NY, USA) for 1 h. at room temperature, and probed with anti-FN antibody followed by an Alexa-Fluor 588-conjugated goat anti-rabbit-IgG. Cells were viewed using an inverted fluorescence microscope (Nikon Eclipse TE 300, Melville, NY, USA). Images were captured using an Exi Blue spot camera (QImaging, Surrey, BC, Canada) connected to a Macintosh computer equipped with iVision (BioVision Technologies, Exton, PA, USA) image analysis software. Cells were semi-permeabilized using 0.2% Triton-X-100 and nuclear staining was performed using DAPI.

### 4.4. CALR siRNA Transfection

Human CALR siRNA and Universal Scrambled Negative control siRNA were purchased from Origene (Cat#SR300567, Origene, Rockville, MD, USA). Transfection was by Nuclefection (Amaxa Biosystems Inc., Gaithersburg, MD, USA). Basic nucleofector kit for primary Mammalian Glial cells (Cat#VPI-1006, Lonza, Allendale, NJ, USA) was purchased and optimized for nucleofection of GBM cells according to manufacturer’s recommendation. Then, 2–3 × 10^6^ cells were suspended in 100 μL Basic Nucleofector^TM^ solution and mixed with 300 nM (30 pmol/sample) CALR siRNA or Universal scrambled negative control siRNA. The cell-siRNA mixtures were then transferred to a Lonza-certified cuvette and subjected to nucleofection using a pre-existing program (T-020) on the nucleofector device. Immediately after nucleofection, 500 μL of the culture medium was added to cells in the cuvette, and cells were transferred to a culture dish containing complete growth medium and incubated in a humidified CO_2_ incubator at 37 °C. Transfection efficiency was assessed using pMax-GFP. CALR-knockdown was confirmed by immunoblot analysis.

### 4.5. CALR and α5-Integrin cDNA Transfection

Mammalian expression plasmids mEmerald-Calreticuli-N-16 and the empty vector control plasmid were a gift from Michael Davidson (Addgene, plasmid#54023). DNA transfection was performed using Lipofectamine 3000 Reagent (Cat#L3000001, Life Technologies, Waltham, MA, USA) following manufacturer’s protocol. GBM cells washed once in PBS and plated in either 24-well plates for immunofluorescence analysis or 6-well plates for FNMA. Then, 500–2500 ng of plasmid DNA and 1.5–7.5 μL of Lipofectamine 3000 Transfection reagent respectively were diluted in 25–125 μL of Opti-MEM medium (ThermoFisher Scientific, Grand Island, NY, USA). The diluted DNA was then added to the diluted Lipofectamine 3000 reagent (1:1 ratio), mixed gently and incubated for 5 min. at room temperature. Following incubation, the DNA-lipid complex was added to cells and incubated for 1–2 days at 37 °C in a humidified CO_2_ incubator. Cells were later selected based on Neomycin resistance with Geneticin (G418, 600 μg/mL) for few weeks and then sorted by FACS using GFP. Stable GBM-2 and GBM-3 cell lines expressing both calreticulin and the empty vector backbone were established. Non-transfected cells and/or empty vector transfected cells were used as controls. pcDNA 3.1 (X5C5) chimeric integrin cDNA construct containing the complete human α5 cDNA [25] was transfected into GBM cells by Lipofectamine as described above. pcDNA3.1 empty vector was used as a negative control for transfection. DOC soluble and insoluble lysates from transiently transfected cells grown for 24 h were later tested for both α5 integrin and FNMA expression, respectively.

### 4.6. Compaction Assay

Hanging drops were generated as previously described in [49] and incubated under tissue culture conditions for 24 h., whereupon images of cell sheets were captured and digitized. Image analysis was performed using IPLab imaging software. Each image was adjusted for optimum contrast and outlines were automatically traced. The number of pixels within the outlines were calculated by the imaging software and converted to area in mm^2^. Data points representing the mean and standard error for aggregate surface area expressed in mm^2^ were calculated from 10–15 aggregates of each cell line.

### 4.7. Dispersal Velocity Assay

Aggregates from all lines were generated by the hanging drop method as described in [49]. Single aggregates were plated into each well of a 12-well tissue culture plate containing 2 mL of pre-warmed CO_2_-independent medium. Images of aggregate spreading was captured every hour for 8 h and analyzed in ImageJ by measuring change in aggregate diameter as a function of time. Data was plotted and the velocity of aggregate spreading determined by linear regression analysis. Only slopes with an *r*^2^ value of 0.95 and above were used to calculate dispersal velocity. Data was normalized with initial aggregate diameter. Twelve aggregates were used to generate an average dispersal velocity and attendant standard deviations and standard errors.

### 4.8. Ex Vivo Mouse Retina and Brain Slice Dispersal Assays

This study was carried out in strict accordance with the recommendations in the Guide for the Care and Use of Laboratory Animals of the National Institutes of Health. All work on mice was performed under an approved Rutgers Institutional Animal Care and Use Committee (Rutgers-IACUC) under protocol, #16-002. Euthanasia was performed using CO_2_ inhalation followed by cervical dislocation as per AVMA and Rutgers IACUC guidelines. Retinas were dissected as previously described [50]. Briefly, mice were sacrificed by CO_2_ inhalation followed by cervical dislocation. Eyes were removed and fixed in 4% paraformaldehyde for 24 h. Retinas were resected, washed in PBS, then incubated in tissue culture medium for several hours prior to seeding with GBM spheroids. Brains were isolated after harvesting of the retinas. Brains were washed three times in 50 mL of ice-cold PBS and then fixed in 10% paraformaldehyde for 24 h. One hundred micrometer–thick brain slices were generated using a Leica VT1000A vibratome. Dissected retinas and brain slices were washed three times in PBS, rinsed with complete tissue culture medium, and incubated for four hours, whereupon 3D spheroids composed of GBM cells labeled with the membrane intercalating dye, PKH67 (Sigma Aldrich, MO, USA) were deposited on the retina or brain slice surface and allowed to spread for 24 h. Spreading was quantified by tracing around the fluorescent signal. Area was calculated as described above.

### 4.9. Statistical Analysis

Relevant data sets were first analyzed for normality using the D’Agostino-Pearson normality test. Those data sets found to be normally distributed were analyzed using the parametric, unpaired, two-sided Student’s *t*-test. Those data for which normality could not be determined, a non-parametric Wilcoxon Rank-Sum test was used. For both methods, a *p*-value < 0.05 was considered significant.

## 5. Conclusions

A pharmacologic approach using Dex is logical inasmuch as it is routinely used as palliative therapy for glioblastoma. Despite its efficacy in reducing edema, side-effects of Dex treatment often limit its long-term use. Accordingly, patients are typically weaned off the drug as soon as clinically possible [45]. Despite reports citing corticosteroid-mediated regression of GBM [51,52], it is widely held that corticosteroid use, particularly concomitant with radiation and chemotherapy, compromises survival of glioblastoma patients [43,44]. Moreover, its effect on global immunosuppression [53] has further contributed to the general perception that high dose, long-term use of Dex may not provide sufficient treatment benefit to GBM patients apart from its role as palliative therapy. Our previous observations on the dispersal and growth inhibitory effects of Dex [1], inspired us to determine whether it may be possible to achieve similar results using strategies that may mitigate the negative side-effects of high-dose, long-term Dex use. The current study suggests that CALR may be a potential target of future research since it appears to act as an intermediary between Dex treatment and restoration of FNMA. An understanding of how CALR activates α5 integrin may provide valuable insight into development of small molecules that can activate fibronectin matrix assembly and attendant inhibition of dispersal while avoiding off-target interactions and their deleterious side-effects.

## Figures and Tables

**Table 1:** Summary table of the kinetics of CALR, InsFn (FNMA), Integrin-5, cohesion, and dispersal in response to Dex treatment.

Treatment	α5 Integrin Expression	CALR Expression	FNMA	Cohesion	Dispersal
VC	Figure 2		Figure 2		
X5C5	Figure 2		Figure 2		
No Dex	[1]	Figure 3B	[1]	[1]	[1]
Dex	[1]	Figure 3B	[1]	[1]	[1]
Dex + CALR siRNA			Figure 4A,B		
Dex + SC			Figure 4A,B		
VC		Figure 5A,B	Figure 5A,B	Figure 6A,B	Figure 6C–E
CALR		Figure 5A,B	Figure 5A,B	Figure 6A,B	Figure 6C–E

VC = vector control, X5C5 = wild-type α5 integrin expression vector, SC = scrambled SiRNA control, CALR siRNA = human CALR siRNA vector; CALR = calreticulin/GFP fusion vector. Red denotes low, green denotes high.

## Abbreviations

CALR	Calreticulin
CHO	Chinese Hamster Ovary
CIL	Contact inhibition of locomotion
DAPI	4′,6-diamidino-2-phenylindole
Dex	Dexamethasone
DOC	Deoxycholate
DV	Dispersal velocity
ECM	Extracellular matrix
ER	Endoplasmic reticulum
ERK	Extracellular signal-regulated kinase
FACS	Fluorescence activated cell sorter
FN	Fibronectin
FNMA	Fibronectin matrix assembly
GAPDH	Glyceraldehyde 3-phosphate dehydrogenase
GBM	Glioblastoma
GFP	Green fluorescence protein
InsFN	Insoluble fibronectin matrix
MEM	Minimal essential medium
NHA	Normal human astrocytes
PBS	Phosphate buffered saline
siRNA	small interfering RNA
TCGA	The Cancer Genome Atlas
